# Surveilling COVID-19 Emotional Contagion on Twitter by Sentiment Analysis

**DOI:** 10.1192/j.eurpsy.2021.3

**Published:** 2021-02-03

**Authors:** Cristina Crocamo, Marco Viviani, Lorenzo Famiglini, Francesco Bartoli, Gabriella Pasi, Giuseppe Carrà

**Affiliations:** 1 Department of Medicine and Surgery, University of Milano-Bicocca, Monza, Italy; 2 Department of Informatics, Systems, and Communication, University of Milano-Bicocca, Milano, Italy; 3 Department of Mental Health & Addiction, ASST Nord Milano, Bassini Hospital, Cinisello Balsamo, Milano, Italy; 4 Division of Psychiatry, University College London, 149 Tottenham Court Road, London, United Kingdom

**Keywords:** Emotional contagion, mental health, sentiment analyses, social media

## Abstract

**Background:**

The fight against the COVID-19 pandemic seems to encompass a social media debate, possibly resulting in emotional contagion and the need for novel surveillance approaches. In the current study, we aimed to examine the flow and content of tweets, exploring the role of COVID-19 key events on the popular Twitter platform.

**Methods:**

Using representative freely available data, we performed a focused, social media-based analysis to capture COVID-19 discussions on Twitter, considering sentiment and longitudinal trends between January 19 and March 3, 2020. Different populations of users were considered. Core discussions were explored measuring tweets’ sentiment, by both computing a polarity compound score with 95% Confidence Interval and using a transformer-based model, pretrained on a large corpus of COVID-19-related Tweets. Context-dependent meaning and emotion-specific features were considered.

**Results:**

We gathered 3,308,476 tweets written in English. Since the first World Health Organization report (January 21), negative sentiment proportion of tweets gradually increased as expected, with amplifications following key events. Sentiment scores were increasingly negative among most active users. Tweets content and flow revealed an ongoing scenario in which the global emergency seems difficult to be emotionally managed, as shown by sentiment trajectories.

**Conclusions:**

Integrating social media like Twitter as essential surveillance tools in the management of the pandemic and its waves might actually represent a novel preventive approach to hinder emotional contagion, disseminating reliable information and nurturing trust. There is the need to monitor and sustain healthy behaviors as well as community supports also via social media-based preventive interventions.

## Introduction

« *When you say something on Twitter, it is “peer reviewed” by thousands … not a select few.* ».

C. Michael Gibson, Harvard Medical School (Twitter 02.19.2020).

SARS-CoV-2 was first reported from Wuhan, China, on December 31, 2019. Having ascertained person-to-person transmission, the World Health Organization (WHO) declared the outbreak of the related respiratory illness to be a global health emergency on January 30, 2020 [1]. While scientists were struggling to expand scientific knowledge on the pathogen’s spread, biology, and variable clinical manifestations (from asymptomatic to mild and severe), social media were at the forefront of the information challenge, since an “infodemic” of rumor, myth, and misinformation might have had a role in hindering both the disease control and its containment. Posts related to this global emergency have been impressively growing as a trend topic on Twitter, with unverified sources of information generating the most extreme statements, far more amplified and pervasive than the measured institutional messages from the WHO and the Centers for Disease Control and Prevention [2]. Worldwide, there seems to be a rapidly evolving debate in which everyone shares her/his subjective perspectives (either positive or negative), mixed-up with legitimate and authoritative sources of information. This situation may engender the echo chamber phenomenon on social media (i.e., people are likely to hear and share opinions that are similar to their own, due to the homophily property characterizing social environments) [3], leading to *community anxiety* and *emotional contagion.* Emotional contagion may occur via several mechanisms, including *mimicry* (emotional expression activates synchronous behavior), *category activation* (exposure primes specific emotional categories and processes), and *social appraisal* (emotions of other subjects guide our own emotion appraisals) [4]. Daily tweets content may impact on individuals’ sentiment, by spreading online emotions, leading people to experience the same emotions without their awareness [5,6].

A timely evaluation of the sentiment and emotional contagion on social media might be useful to both deal with risky communication and inform potential preventive strategies. We assumed that content trends on Twitter have been marked worldwide by some major key events about the outbreak. These have certainly included at least: (a) the first WHO report (January 21, 2020); (b) the WHO declaration of Public Health Emergency of International Concern (January 30, 2020); (c) the Diamond Princess cruise ship lockdown in Japan (February 4, 2020); and (d) the naming of the new disease (COVID-19, February 11, 2020). In addition, since February 23, 2020, a rapid increase in cases of COVID-19 has been reported in the European Region.

The current study aimed to examine the flow and content of tweets, capturing COVID-19 core discussions and trends following relevant key events. We assumed that some of these key events might have influenced the flow and contents of tweets on COVID-19 [7]. The potential of social media like Twitter in capturing pandemic-related sentiment may ultimately inform novel surveillance methods of individual emotional states and their spread. To this purpose, we gathered contents on Twitter related to COVID-19 between January 19 and March 3, 2020.

## Methods

Based on representative data freely available from Twitter, tweets were gathered using Advanced Programming Interfaces (APIs) as a structured, albeit limited, access point to Twitter’s archives to search for information according to identified criteria (i.e., specific hashtags and keywords). To gather relevant tweets in the selected period, we performed a focused crawling by employing both the hashtag #coronavirus and the keyword “coronavirus.” No additional hashtags or keywords were selected since, in the initial phase, “coronavirus” was the most frequently used word to discuss the topic, and the term COVID-19 was introduced at a later time. Data were preliminarily analyzed through the Language detection library, from Google’s language-detection tool. To elicit both the relevant sentiment and its change in time in relation to the identified key events from discussions on social media, text, and individual-level characteristics about posting users were analyzed, identifying both single tweets (by id) and associated individual users (by username).

These analyses were also carried out considering the classification of users according to the average number of pandemic-related tweets that each of them posted during the selected period (threshold of three). Active users, as compared with inactive ones, were identified as those who were more likely to steer the core discussions and/or to be involved in emotional contagion, as they tweeted much more often than inactive users [8]. Active users may include organizations, bots, and human users. Specific analyses were thus carried out with respect to this additional classification. Those tweets likely belonging to broadcasters and institutional accounts were detected from the discussions of active users, using the Twitter’s API to download metadata associated with the username in the gathering phase. This was achieved by considering Twitter’s *verified* accounts among active users (in Twitter, these accounts are identified by an associated “blue badge”).

Considering the varying usage and the context-dependent meaning of terms in the text, the emotion-specific features related to the sentiments expressed in tweets were decoded by performing: (a) Sentiment analysis, by using both the Valence Aware Dictionary for sEntiment Reasoning (VADER) [9] and the CT-BERT: Covid Twitter BERT (Bidirectional Encoder Representations from Transformers) model [10,11]; and (b) Emotional Analysis, based on the NRC Word-Emotion Association Lexicon (EmoLex), developed by the Canadian National Research Council [12]. Thus, in order to provide an overview on the likely ability of Twitter as a tool to assess and surveil emotional contagion about COVID-19 pandemic, we followed two complementary approaches. The first was based on a fairly computationally inexpensive lexicon-based method, that is widely employed for general-purpose Sentiment Analysis [13]. The second followed a properly trained and fine-tuned semantic-based model allowing to consider the context of the words embedded in tweets specifically within the COVID-19 scenario.

Concerning VADER lexicon-based approach for Sentiment Analysis, it employs a human-generated English sentiment lexicon, where lexical features (i.e., words) are labeled according to their semantic orientation as positive, negative, or neutral, also expressing the sentiment intensity for each lexical feature. In addition, VADER manages a proper handling of punctuation (e.g., the exclamation point (!) increases the magnitude of the intensity without modifying the semantic orientation, e.g., *Coronavirus will not stop us!*), as well as capitalization (ALL-CAPS is used to emphasize a sentiment-relevant word in the presence of other non-capitalized words, *Coronavirus WE WILL WIN THIS TOGETHER*), degree modifiers (which impact on sentiment by either increasing or decreasing its intensity, e.g., I am *extremely* worried about Coronavirus), sentiment-laden slang words, and emoticons/emoji. Furthermore, Stata release 15 (StataCorp, College Station, Texas) was used for additional analyses.

By means of VADER, the assessment of tweets polarity was performed by computing the so-called polarity *compound score* for each tweet. The compound score is computed as the sum of all lexicon ratings associated with words, normalized between −1 (extremely negative) and +1 (extremely positive). Based on the compound scores obtained, we computed the proportion of the resulting negative (compound score ≤ −0.05), neutral (compound score between −0.05 and 0.05), and positive (compound score ≥ 0.05) tweets for the considered time period (January 19–March 3, 2020).

Considering the same tweets and time period, we employed the semantic-based BERT model [14], pretrained on a corpus of messages from Twitter about COVID-19, that is, CT-BERT [10], to perform Sentiment Analysis. The original CT-BERT model—trained on 22.5 million tweets collected between January and April 2020 and containing at least one keyword among “wuhan,” “ncov,” “coronavirus,” “covid,” or “sars-cov-2”—was adapted by adding a so-called *fine-tuning layer* (i.e., a single neural layer) to the model, trained on the SemEval-2017 Task 4 (Sentiment Analysis in Twitter) dataset [15]. This enabled to disambiguate, in the specific COVID-19 context, the neutrality of tweets originally identified by lexicon-based approaches as positive, possibly obtaining greater precision.

The trends of sentiment polarity were evaluated over time, by considering the two different populations from the clusters of Twitter’s users who generated the tweets (active and inactive users). Ratios between both negative and neutral, and negative and positive tweets, were also computed.

Finally, concerning the use of EmoLex, an English annotated lexicon obtained by crowdsourcing (i.e., by means of Amazon’s Mechanical Turk) [12], several emotions including anger, fear, anticipation, trust, surprise, sadness, joy, and disgust, were recognized, detected, and analyzed.

## Results

Between January 19 and March 3, 2020, we gathered 6,065,580 tweets, of which 3,308,476 (82,166 on average per day) written in English (the remaining 45% were in different languages: Spanish 19%, Portuguese 6%, Italian 6%, Others 13%). Considering only the tweets written in English, a total of 1,292,355 unique users were identified, of whom 177,264 (13.7%) posted more than the average number (three) of tweets per user in the selected period.

**Figure 1.**
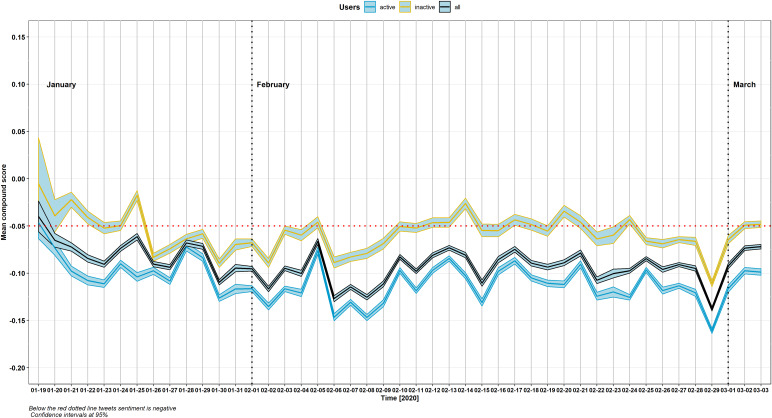
Tweets polarity compound score (January 19–March 3, 2020).

**Figure 2.**
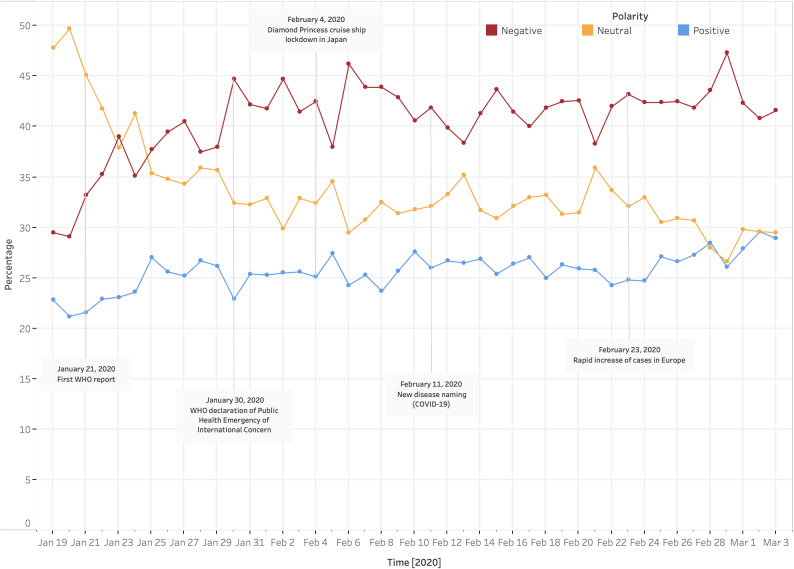
Proportion of tweets by VADER sentiment polarity (January 19–March 3, 2020).

The sentiment about COVID-19 evaluated by means of VADER was on average negative according to the estimated polarity compound scores and relevant 95% Confidence Intervals (Figure 1). Daily volumes (Figure 2) showed a proportion of negative polarity tweets ranging from 28 to 47%, with a polarity ratio—between negative and both neutral and positive tweets—backing negative tweets. Since the first WHO report, the negative sentiment proportion gradually increased over time. However, data dispersion suggested that negative sentiment was likely to swing over time. Several spikes were found across the flow, with appreciable amplifications of negative sentiment occurring following each of the considered key events in time (January 30; February 4; February 11; February 23). This trend was confirmed also in early March when the outbreak in Europe rapidly evolved. Many tweets were posted, between January 19 and March 3, 2020, by a relatively low number of extremely active users, who were more likely to show a negative sentiment as compared with inactive users (blue and yellow lines respectively in Figure 1).

**Figure 3.**
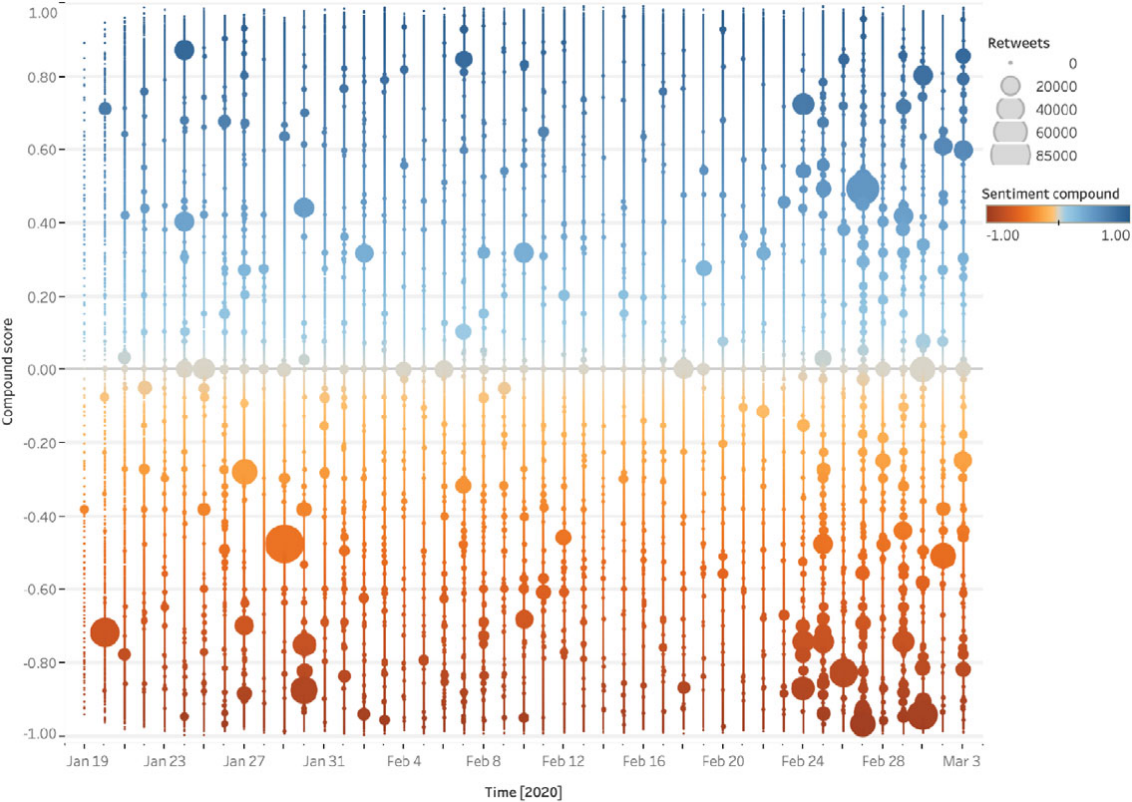
Sentiment and retweets: active users (January 19–March 3, 2020).

Sentiment scores were increasingly negative among active users (dark red bubbles in Figure 3), who were also more likely to immediately retweet both positive and negative tweets, in particular after the increase of cases in Europe (Figure 3).

The trend of tweets evaluated as connoted by a negative sentiment was supported by the semantic approach based on CT-BERT, though a relatively small number of tweets detected as negative by VADER were classified as neutral by CT-BERT. On the other hand, the use of CT-BERT, as compared with the VADER lexicon-based approach, showed a different behavior regarding the attribution of positive and neutral sentiment to tweets. Many of the tweets assessed by VADER as positive were defined as neutral using CT-BERT (Supplementary Figures S1 and S2). These tweets probably encompassed measured messages from institutional accounts as previously described. From Twitter’s *verified* accounts including institutional users CT-BERT detected a proportion of tweets classified as positive of 4.2%, as negative of 19.3%, and as neutral of 76.3%, compared with 26.7, 46.4, and 26.7%, respectively, using the VADER approach.

Finally, the emotional analysis showed contrasting trends, with higher levels of fear (ranging from 18 to 25%) as compared with trust (ranging from 14 to 19%). Remaining emotions did not show any clear tendency.

## Discussion

This is the first study exploring the role of topical news on Twitter’s users as regards the spread of emotional contagion about COVID-19 outbreak. We were able to uncover this effect by considering several dimensions that might suggest some novel preventive approaches involving social media.

### Polarity

A progressively increasing negative polarity characterized the longitudinal perception of Twitter’s users about COVID-19 across the selected key events in time. Rising emotional contagion might represent a warning on different stressors affecting psychological wellbeing during the outbreak. These are likely to include infection fears, frustration, boredom, inadequate provision of information, financial loss, stigma, social distancing up to complete lockdown [16]. It might be argued that Twitter’s users changed their polarity towards the topic since they increasingly realized some characteristics of the COVID-19 outbreak. These would possibly include the unknown risk of being infected and infecting others, based on the uncertainty about asymptomatic status, and the presence of symptoms common to other health problems that might be mistaken for COVID-19. In addition, social distancing measures might have affected psychological wellbeing of vulnerable individuals, if caregivers are far away and other care and support are not in place [17].

### Sentiment Analysis

Since late January, sentiment became more intensively negative following an increase in COVID-19 media coverage which was expected. This change in sentiment is likely to be explained by misinterpretations of factors like risk communication and perception, both over- and under-estimated, about COVID-19 [16,18]. Variations in perception may thus occur, depending on individual background, resilience, and attitudes [19]. However, because of the uncertain nature of the spread, scope, and impact of the disease, emotional distress may affect even those not directly exposed [20]. In addition, poor understanding, perplexity, and confusion may turn into anger if people feel they were exposed to the disease because of others’ negligence [21].

### Public Health Implications

We found several fluctuations over time in trajectories of negative polarity and sentiment for COVID-19, after the release of news on key events. These trends seem attributable to a limited number of very active users, with the remaining large majority on Twitter potentially exposed to emotionally unstable perceptions. However, the evaluation of connections between users is needed to complement sentiment trajectories in a cutting-edge approach to identify characteristics of subjects who may be engaged in critical influences and consider them as the potential target of preventative interventions. This is sharply related to both individual- and area-level components that characterize social networks, with varying size and homogeneity degree between individuals belonging to the same network. It is crucial to better understand how the information differentially spreads across social media.

In terms of emotions, fear exceeds trust since the emotional contagion outlasts under the always-on conditions of Twitter and 24-h news. This supports suggestions about a COVID-19 post-truth scenario based on the celebrity, political party, or intuition of speaker and listener, rather than of best available scientific evidence [22].

A semantic-based model underlying the CT-BERT approach showed that institutional users likely posted tweets classified as neutral. Considering the long-lasting scenario [23-25], there is the need to surveil and sustain healthy behaviors as well as community and family supports to reduce loneliness and psychological isolation in order to maintain public health advice rooted in truth rather than in contested values and preferences [17,20,22]. We henceforth speculate on the opportunity that selected and reliable sources of information like government authorities, aware about relevant rumors [19], may timely provide on social media like Twitter repeated, focused, understandable, and culturally appropriate contents, confronting sentiment fluctuations and nurturing trust and clarity [26]. In order to face this public health crisis, as well as public confusion and fear, clear messages and honest information by governments are needed following expert panels opinions [2]. Consistently, influential individuals like the former US President Barack Obama (about 114.5 million followers on Twitter), have been willing to break silence during pandemic, becoming more engaged on social media to promote safety measures and share stories of inspiring people and organizations [27]. Digital platforms and social media may play an important role in hindering the emotional contagion and in enhancing connectedness of individuals even while in quarantine. These contents can help make people feel less stressed, using focused communication strategies, increasing trust and adherence to behavioral measures [28,29]. These should take into account emotional biases that may act as a barrier in understanding both health information and the need for severe measures such as lockdown and social distancing.

Our preliminary findings are consistent with recent research suggesting the importance of integrating social media as a critical surveillance tool in managing the current evolving pandemic. A better understanding of sentiment trajectories and of how information spreads and individuals interact is needed in order to enable a culture of preparedness, that would help citizens to deal with science-based information, improving bidirectional trust between community and authorities [30,31]. However, in the current study, we were able to take into account just individual-level components of emotional contagion. We could not identify area-level characteristics of subjects who may be engaged in emotionally unstable connections. Therefore, future research, in order to better grasp Twitter’s promising surveillance properties, should incorporate both semantic approaches and network analyses that will provide details about users’ interactions.

To design an integrated multilevel surveillance tool grounded on this novel source of information, we are in need of a systematic and continuous collection, collation, and analysis of data benefiting from different methods, and the timely dissemination of reliable and trustworthy information. This would possibly allow to offer a tool able to mitigate the sentiment fluctuations in Twitter’s global stream of data and to promote individuals’ emotional wellbeing during and after a possible lockdown, rooting out social causes of post-truth.

As part of the public health response to COVID-19, a Twitter-based surveillance of the emotional contagion seems viable, and relevant preventive activities via social media are probably needed.

## Data Availability

Data that support the findings of this study are freely available from Twitter through Advanced Programming Interfaces (APIs).

## Supplementary material

For supplementary material accompanying this paper visit https://doi.org/10.1192/j.eurpsy.2021.3. click here to view supplementary material
