# Supplementary material for: Surveilling COVID-19 Emotional Contagion on Twitter by Sentiment Analysis
Source: Eur Psychiatry. 2021 Feb 3;64(1):e17. doi: 10.1192/j.eurpsy.2021.3 (PMC7943954; doi:10.1192/j.eurpsy.2021.3)
Supplement: Supplementary file 1 [file S0924933821000031sup001.docx]

**Surveilling COVID-19 emotional contagion on Twitter**

**- *Sentiment analyses –***

***Supplementary material***

**Figure S1. Sentiment of tweets: VADER classification (number of tweets)**

**
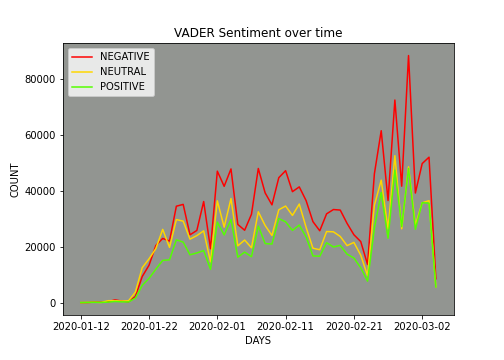
**

**(a)**

**
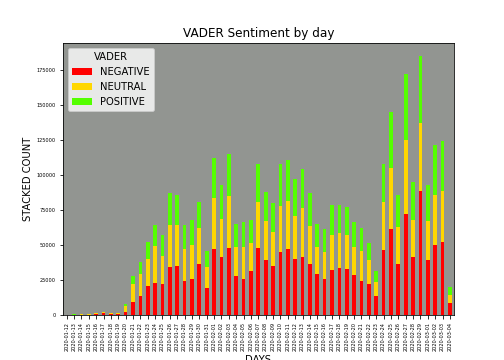
**

**
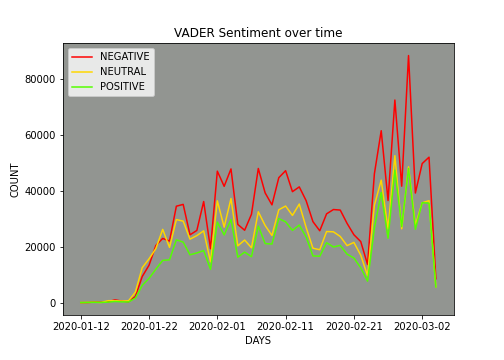
**

**(b)**

(a) count of tweets; (b) stacked count of tweets.

Proportions of sentiment of tweets using VADER: Negative: 42.1%; Neutral: 31.6%; Positive: 26.2%

**Figure S2. Sentiment of tweets: CT-BERT classification (number of tweets)**

**
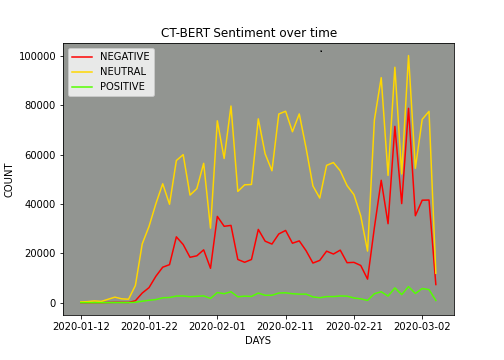
**

**(a)**

**
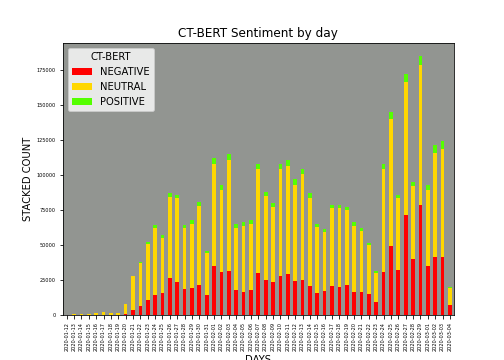
**

**
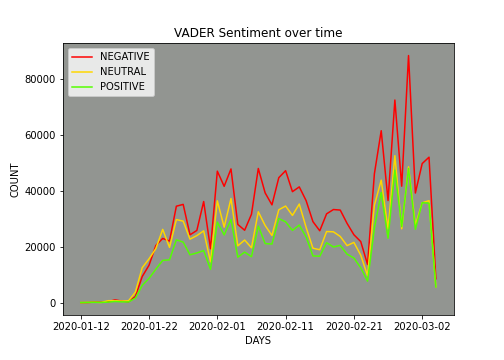
**

**(b)**

(a) count of tweets; (b) stacked count of tweets.

Proportions of sentiment of tweets using CT-BERT: Negative: 29.9%; Neutral: 66.8%; Positive: 3.4%
